# Evaluation of a computer-aided diagnostic model for corneal diseases by analyzing *in vivo* confocal microscopy images

**DOI:** 10.3389/fmed.2023.1164188

**Published:** 2023-04-20

**Authors:** Yulin Yan, Weiyan Jiang, Yiwen Zhou, Yi Yu, Linying Huang, Shanshan Wan, Hongmei Zheng, Miao Tian, Huiling Wu, Li Huang, Lianlian Wu, Simin Cheng, Yuelan Gao, Jiewen Mao, Yujin Wang, Yuyu Cong, Qian Deng, Xiaoshuo Shi, Zixian Yang, Qingmei Miao, Biqing Zheng, Yujing Wang, Yanning Yang

**Affiliations:** ^1^Department of Ophthalmology, Renmin Hospital of Wuhan University, Wuhan, Hubei Province, China; ^2^Department of Gastroenterology, Renmin Hospital of Wuhan University, Wuhan, Hubei, China; ^3^Department of Resources and Environmental Sciences, Resources and Environmental Sciences of Wuhan University, Wuhan, Hubei Province, China; ^4^Department of Ophthalmology, Zhongnan Hospital of Wuhan University, Wuhan, Hubei, China

**Keywords:** deep learning, IVCM, ophthalmology, cornea, artificial intelligence

## Abstract

**Objective:**

In order to automatically and rapidly recognize the layers of corneal images using *in vivo* confocal microscopy (IVCM) and classify them into normal and abnormal images, a computer-aided diagnostic model was developed and tested based on deep learning to reduce physicians’ workload.

**Methods:**

A total of 19,612 corneal images were retrospectively collected from 423 patients who underwent IVCM between January 2021 and August 2022 from Renmin Hospital of Wuhan University (Wuhan, China) and Zhongnan Hospital of Wuhan University (Wuhan, China). Images were then reviewed and categorized by three corneal specialists before training and testing the models, including the layer recognition model (epithelium, bowman’s membrane, stroma, and endothelium) and diagnostic model, to identify the layers of corneal images and distinguish normal images from abnormal images. Totally, 580 database-independent IVCM images were used in a human-machine competition to assess the speed and accuracy of image recognition by 4 ophthalmologists and artificial intelligence (AI). To evaluate the efficacy of the model, 8 trainees were employed to recognize these 580 images both with and without model assistance, and the results of the two evaluations were analyzed to explore the effects of model assistance.

**Results:**

The accuracy of the model reached 0.914, 0.957, 0.967, and 0.950 for the recognition of 4 layers of epithelium, bowman’s membrane, stroma, and endothelium in the internal test dataset, respectively, and it was 0.961, 0.932, 0.945, and 0.959 for the recognition of normal/abnormal images at each layer, respectively. In the external test dataset, the accuracy of the recognition of corneal layers was 0.960, 0.965, 0.966, and 0.964, respectively, and the accuracy of normal/abnormal image recognition was 0.983, 0.972, 0.940, and 0.982, respectively. In the human-machine competition, the model achieved an accuracy of 0.929, which was similar to that of specialists and higher than that of senior physicians, and the recognition speed was 237 times faster than that of specialists. With model assistance, the accuracy of trainees increased from 0.712 to 0.886.

**Conclusion:**

A computer-aided diagnostic model was developed for IVCM images based on deep learning, which rapidly recognized the layers of corneal images and classified them as normal and abnormal. This model can increase the efficacy of clinical diagnosis and assist physicians in training and learning for clinical purposes.

## 1. Introduction

The cornea is located in the outermost layer of the eye, and as the first step in vision formation, its transparency and refractive index allow light to be refracted into the eye and focused on the retina. Therefore, structural and functional damage at any level of the cornea may lead to vision loss or even blindness (1). Corneal diseases, such as granular corneal dystrophy and Fuchs’ endothelial corneal dystrophy, may damage a single layer of the cornea, while infectious keratitis, corneal mechanical damage, and chemical injuries to the eye may cause multi-layer or even whole corneal damage (2). Meanwhile, systemic diseases, such as diabetes mellitus and rheumatoid arthritis, also affect the cornea (3). Corneal blindness is the fourth most common cause of blindness in the world (4), which affects more than 5% of the world’s blind population and mainly influences marginalized populations (5), while according to the estimation of the World Health Organization (WHO), nearly 80% of corneal blindness is avoidable.

*In vivo* confocal microscopy (IVCM) is a non-invasive imaging tool that facilitates observation of the cornea and its structural changes at the cellular level in regular and pathological states, and it possesses the advantages of real-time, non-invasive, repeatable, and high-resolution (6). At present, it is being used increasingly in clinical practice, and it is an important reference for the clinical diagnosis of several corneal diseases. The monitoring of corneal microstructural changes is also advantageous to optimize the targeted management of keratoconus (7) and to assess the prognosis of patients with systemic diseases (8). In clinical practice, however, due to the small area of the IVCM lens (400 μm × 400 μm per frame), detailed evaluation of the cornea requires the acquisition of a large number of images (typically 50–300 images per eye), while manual analysis of the IVCM images is extremely labor-intensive, time-consuming, and is inherently subjective (9), and IVCM reading also requires a certain level of experience, in which a certain training period is necessary for physicians to distinguish various layers of corneal images and their performance. With the shortage of ophthalmologists in both developed and developing countries (10), improving the accuracy and diagnostic efficiency of IVCM image reading in the identification of corneal layers and their performance can reduce clinical and scientific workload and improve physicians’ efficiency.

Advances in artificial intelligence (AI) are transforming screening, diagnosis, and treatment in all areas of medicine (11), and the application of AI to ophthalmic diseases has also significantly evolved over the past decade. To date, AI has made significant breakthroughs in the segmentation, quantification, and identification of corneal epithelial cells, corneal nerves, corneal endothelial cells, fungal hyphae, dendritic cells, and inflammatory cells in IVCM images (9, 12–15), and it has demonstrated an excellent performance in terms of speed and accuracy of film reading, which can make healthcare more accessible and cost-effective. However, no relevant study has yet evaluated multilevel corneal IVCM images. The present study aimed to develop an AI-assisted automatic diagnostic model for IVCM images, improve the speed of diagnosing corneal diseases and the detection rate of abnormal corneal images, reduce physicians’ workload, and assess its efficacy in clinical application and the possibility of facilitating the intelligent screening of corneal diseases.

## 2. Methods

### 2.1. Datasets and preprocessing

This study retrospectively collected corneal images from patients who underwent IVCM at the Renmin Hospital of Wuhan University (Wuhan, China) and Zhongnan Hospital of Wuhan University (Wuhan, China) from January 2021 to August 2022. All IVCM images were acquired by senior ophthalmic confocal microscopists with more than 15 years of experience through strictly standardized operation using IVCM (HRT III/RCM Heidelberg Engineering, Germany), and images were anonymously processed before labeling and model training. The study was conducted following the Declaration of Helsinki, which was approved by the Ethics Committee of Renmin Hospital of Wuhan University (Approval no. WDRY2021-K148), and the need for informed consent was waived due to the retrospective design of the study.

**Figure 1:**
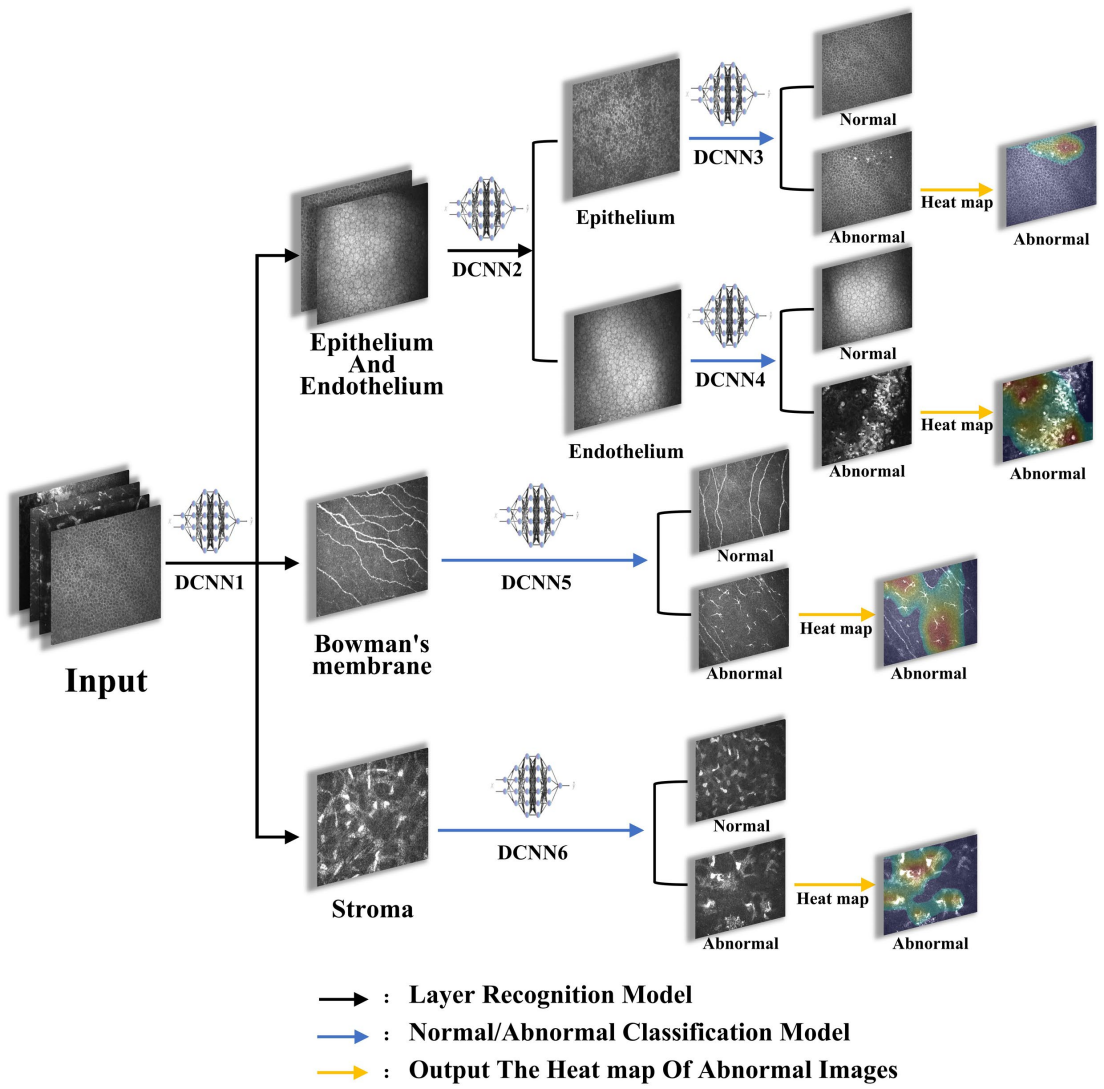
The flowchart of the model. Images were imported into the proposed architectures, initially sorted by DCNN1 to recognize epithelium/endothelium, bowman’s membrane, and stroma, and were then sorted by DCNN2 to recognize epithelium and endothelium. Afterwards, images of each layer were classified to abnormal and normal by DCNN3, DCCN4, DCCN5, and DCCN6. Finally, the heat map of abnormal images was plotted.

All images were screened by professional ophthalmologists to eliminate low-quality images due to overexposure, insufficient light, poor focus, blurred shooting or poor contact, etc. (These images account for 5.27% of the total number of the images we collected). A total of 18,101 images of 314 patients from the Eye Center of Wuhan University People’s Hospital and 1,510 images of 109 patients from Wuhan University Central South Hospital were finally included. Supplementary Table S1 shows the baseline information and sample distribution and Supplementary Table S2 shows the clinical diagnosis and number of included cases. After extracting the depth information by optical character recognition, the qualified images were first converted to a uniform size of 384 pixels × 384 pixels (corresponding to the size of an IVCM image without any text or borders). In order to classify images in a more standardized way, we established various normal and abnormal image criteria based on the book *In Vivo Laser Confocal Microscopy Atlas Of Cornea 2014*, *Atlas Of Ocular Surface In Vivo Confocal Microscopy 2021* and clinical experience from cornea experts. Normal image criteria: (1) all epithelial cells were observed to be structurally intact and morphologically clear in the normal epithelial images; (2) the background of the normal bowman’s membrane images are homogeneous and moderately reflective, while highly reflective nerve fibers with moderate thickness, curvature and density are visible; (3) normal stroma images are characterized by the absence of a characteristic dark reflective background and well defined stromal cell nuclei, with occasional few coarse highly reflective stromal nerves; (4) normal endothelial images can be seen as regularly arranged uniform 5–7 sided cells with clear cell borders. In order to improve sensitivity to screen out abnormal images as widely as possible, our inclusion criteria for abnormal images include: (1) the abnormal epithelial image shows edema of epithelial cells, unclear structure, enlarged interstitial space and inflammatory cells; (2) abnormal bowman’s membrane images can reveal obvious nerve fiber tortuosity, thinning and reduced density, also more than 10 unactivated Langerhans cells and/or any activated Langerhans cell or oval inflammatory cell can be observed; (3) abnormal stroma images may show stromal cell swelling, activation, pine-needle-like highly reflective scarring and amoebic encapsulation, fungal hyphae, fungal spores, neovascularization, etc.; (4) abnormal endothelial images can appear as endothelial cell swelling, degeneration, dystrophy and the appearance of arbitrary posterior corneal deposits. Images were independently classified by two professional cornea specialists and when the two cornea specialists obtained the same classification result, a basic true label was assigned to each image, and if there was any disagreement, a third cornea specialist with over 15 years of experience made the final decision. Images were first classified into epithelium, bowman’s membrane, stroma, and endothelium classes according to corneal layers, and images of each layer were then re-classified into normal and abnormal classes based on clinical diagnosis. Descemet’s membrane was not involved in the training because its thickness was very low and it was closely attached to the corneal endothelium (16), thus, negligible images were collected. Furthermore, its clinical significance is relatively limited, and it is mainly manifested in the field of corneal transplantation (17). Figure 1 shows the workflow of the model.

### 2.2. Development of the model

A deep learning model based on ResNet-50 module was used for image classification training using early stopping, drop out, and dataset augmentation to reduce the risk of overfitting. A server with four NVIDIA Geforce GTX 2080s devices (8GB GPU memory) was used to train the model, and the algorithms were written in Python (version 3.6.5) with the open source TensorFlow library (version 1.12.2) and Keras library (version 2.2.5) as the backend.

The model training was summarized as follows: (1) deep convolutional neural network 1 (DCNN1): classification of images to epithelium/endothelium, bowman’s membrane, and stroma classes; (2) DCNN2: introducing the image depth information to distinguish the epithelial and endothelial images; (3) DCNN3, DCNN4, DCNN5, and DCNN6: after the layer classification, corneal images of the 4 layers were classified to normal and abnormal classes, respectively. (4) Output image layer information, depth information, and normal/abnormal diagnosis were considered as results, and output heat map results of abnormal images were obtained.

Briefly, after the images were input into the system, they were initially classified into epithelial/endothelial, preelastic, and stroma by the three-classification model DCNN1 based on the image layers, then the epithelial/endothelial images were classified into epithelial and endothelial by the two-classification model DCNN2, finally the images that had been classified into four layers were classified into normal and abnormal for each layer by the corresponding two-classification models DCNN3-6. For the choice of activation function, we mainly use sigmoid in the two-classification model and softmax in the three-classification model.

Images were assigned to the training dataset and the internal test dataset at a ratio of 8:2 in the number of patients, and this process ensures that each classified image is restricted to the appropriate set, avoiding overestimation of model performance due to image mixing and label leakage in which the number of images. In DCNN1 model was 17,521 (training dataset: test dataset =13,276:4245), that of images in DCNN2 model was 4,122 (training dataset: test dataset =2,720:1,402), that of images in DCNN3 model was 2,521 (training dataset: test dataset =1,704:817), that of images in DCNN5 model was 3,607 (training dataset: test dataset =2,059:1,295), that of images in DCNN5 model was 9,792 (training dataset: test dataset =8,497:1,295), and that of images in DCNN6 model was 1,601 (training dataset: test dataset = 1,016:585; Figure 2).

**Figure 2:**
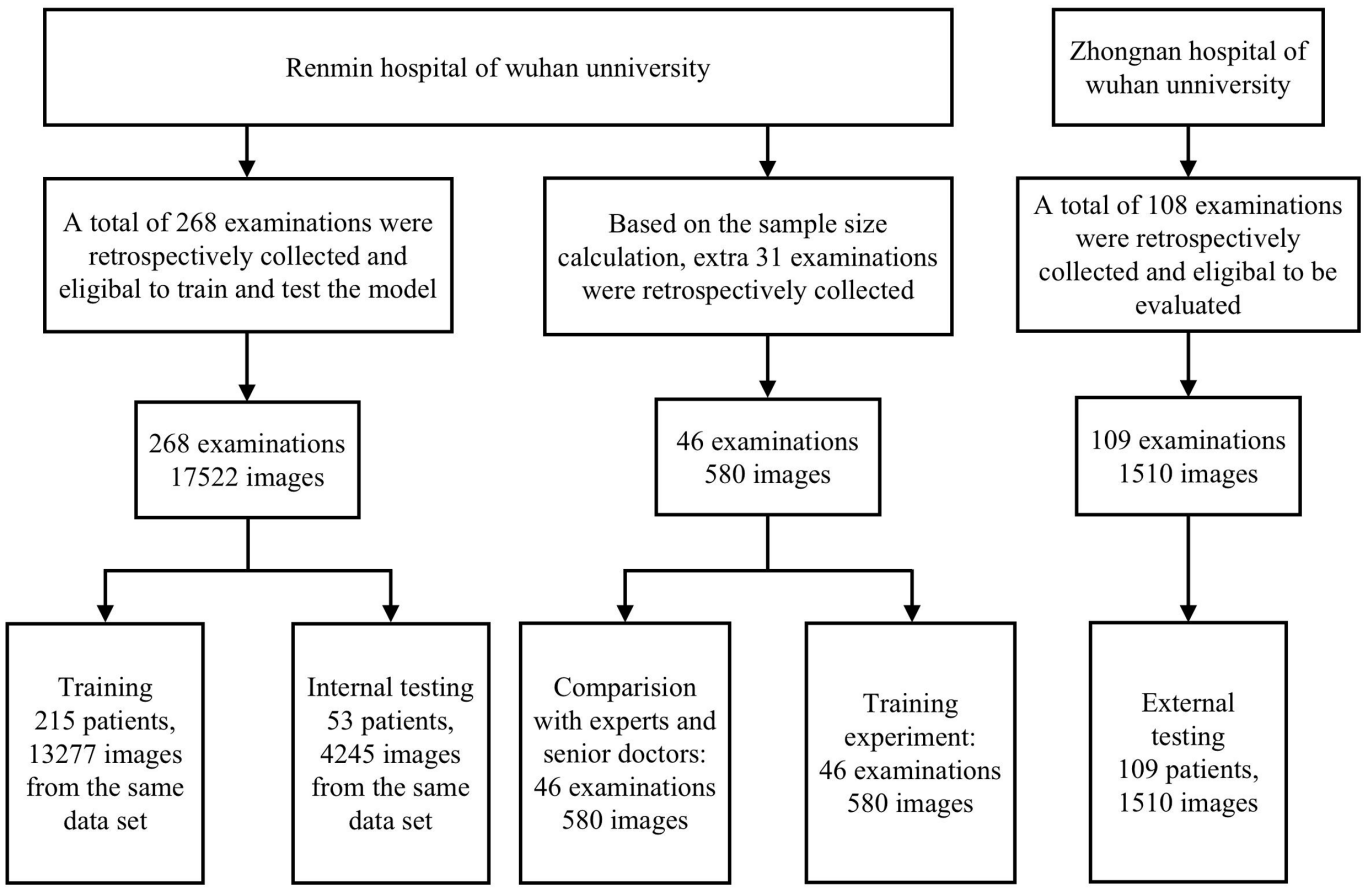
Flowchart of the model development and validation.

### 2.3. Evaluation of the model

A dataset for internal testing (Renmin Hospital of Wuhan University) and a dataset for external testing (Zhongnan Hospital of Wuhan University) were used to evaluate the performance of the model. Accuracy, specificity, sensitivity, receiver operating characteristic (ROC) curve, area under the curve (AUC), positive predictive value (PPV), and negative predictive value (NPV) with 95% confidence interval (CI) were used to assess the performance of the model.

### 2.4. Comparison between the diagnostic performance of DCNNs and ophthalmologists

After the training of the model was completed, 580 images were selected independently of the training dataset and the test dataset, according to a sample of 282 images from the positive group and 282 images from the negative group. A power of 90% could be achieved to detect a difference of 0.1000 between an AUC under the null hypothesis of 0.9500 and an AUC under the alternative hypothesis of 0.9100 using a two-sided *z*-test at a significance level of 0.04000 and the data were discrete (rating scale) responses. Then, two specialists with more than 10 years of experience and two senior physicians with 5–10 years of experience in IVCM were invited to participate in the human-machine competition, they independently diagnosed image levels and normal/abnormal performance, while the same researcher recorded the elapsed time in the test, and the accuracy of the assessment results and the time spent on the assessment between physicians and the model were finally compared.

### 2.5. Comparison the performance of the ophthalmologists with and without model assistance

Using the same batch of 580 images, 8 trainees with no professional IVCM training and less than 3 years of experience were randomly divided into groups A (A1, A2, A3, A4) and B (B1, B2, B3, B4). Trainees in group A first diagnosed and recorded the results of 580 images with model assistance (images were first evaluated by computer and lesion areas were marked in the form of heat maps) separately, after a 2-week washout period, they re-evaluated these 580 images without model assistance and recorded the results. Trainees in group B initially diagnosed and recorded the results of 580 images independently without model assistance, after a 2-week washout period, they re-evaluated these 580 images with model assistance. Finally, the accuracy of the two assessments was compared between groups A and B.

### 2.6. Statistical analysis

SPSS 25.0 (IBM, Armonk, NY, United States) and MedCalc 19.1–64 bit (MedCalc Software Ltd., Ostend, Belgium) software were used for statistical analysis of the data. A *t*-test was utilized to analyze the difference in accuracy between the model and ophthalmologists, and the Mann–Whitney *U* test was applied to compare the accuracy of trainees with and without model assistance to assess the efficacy of the model in clinical application. *p* < 0.05 was considered statistically significant.

## 3. Results

### 3.1. Results in both internal and external datasets

**Figure 3:**
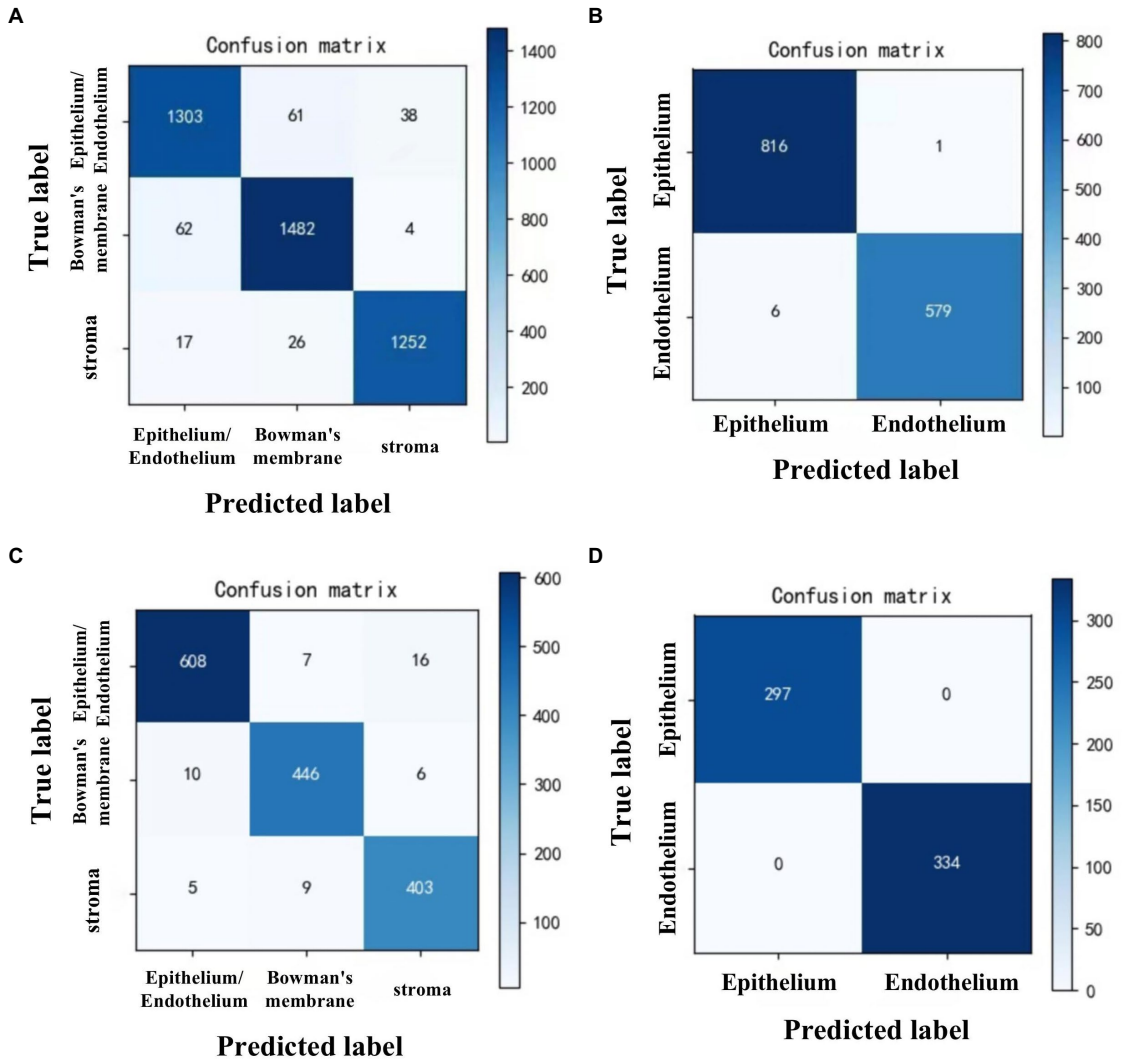
Confusion matrix diagrams of 2 DCNN models in internal and external test datasets. DCNN1: classifying all images to epithelium/endothelium, bowman’s membrane, and stroma classes. DCNN2: distinguish the epithelial and endothelial images. **(A)** DCNN1 in internal test dataset. **(B)** DCNN2 in internal test dataset. **(C)** DCNN1 in external test dataset. **(D)** DCNN2 in external test dataset.

To recognize the epithelium/endothelium, bowman’s membrane, and stroma images, DCNN1 had the highest accuracy of 0.951 (95% CI = 0.945–0.957) in the internal dataset and 0.965 (95% CI = 0.956–0.974) in the external dataset. In distinguishing between epithelium and endothelium images, DCNN2 reached the highest accuracy of 0.995 (96% CI = 0.991–0.999) in the internal dataset and 1.000 in the external dataset. In classifying the abnormal and normal images of each layer, DCNN3, DCNN4, DCNN5, and DCNN6 all achieved the highest accuracy with values of 0.961 (95% CI = 0.948–0.974), 0.932 (95% CI = 0.919–0.944), and 0.945 (95% CI = 0.932–0.957), and 0.959 (95% CI = 943–0.975) in the internal dataset and 0.983 (95% CI = 0.968–0.998), 0.972 (95% CI = 0.957–0.987), 0.940 (95% CI = 0.917–0.963), and 0.982 (95% CI = 0.968–0.996) in the external dataset, respectively. The confusion matrix diagrams of DCNN1 and DCNN2 are shown in Figure 3, and the specific competence of DCNN3–DCNN6 is presented in Table 1. The test results demonstrated that the model had a high potential for classifying and diagnosing multi-category corneal images of IVCM.

**Table 1:** The classification performance of deep convolutional networks.

		Accuracy (95% CI)	Sensitivity (95% CI)	Specificity (95% CI)	PPV (95% CI)	NPV (95% CI)	AUC
DCNN3	Internal	0.961 (0.948–0.974)	0.924 (0.898–0.950)	0.998 (0.993–1.002)	0.997 (0.992–1.003)	0.929 (0.905–0.953)	0.994
	External	0.983 (0.968–0.998)	0.986 (0.966–1.006)	0.981 (0.959–1.003)	0.979 (0.955–1.003)	0.987 (0.969–1.005)	0.997
DCNN4	Internal	0.932 (0.919–0.944)	0.888 (0.866–0.910)	0.975 (0.964–0.986)	0.973 (0.961–0.985)	0896 (0.875–0.917)	0.997
	External	0.972 (0.957–0.987)	0.987 (0.972–1.002)	0.957 (0.930–0.983)	0.958 (0.932–0.984)	0.987 (0.971–1.002)	0.975
DCNN5	Internal	0.945 (0.932–0.957)	0.998 (0.996–1.001)	0.881 (0.855–0.908)	0.908 (0.887–0.929)	0.998 (0.994–1.002)	0.996
	External	0.940 (0.917–0.963)	0.952 (0.923–0.981)	0.928 (0.892–0.963)	0.930 (0.896–0.965)	0.950 (0.920–0.981)	1.000
DCNN6	Internal	0.959 (0.943–0.975)	0.904 (0.865–0.943)	0.992 (0.983–1.001)	0.985 (0.968–1.002)	0.945 (0.922–0.968)	0.979
	External	0.982 (0.968–0.996)	0.975 (0.951–0.999)	0.988 (0.972–1.005)	0.988 (0.970–1.005)	0.977 (0.955–1.000)	0.996

DCNN3, classification model of normal and abnormal epithelium images; DCNN4, classification model of normal and abnormal bowman’s membrane images; DCNN5, classification model of normal and abnormal stroma images; DCNN6, classification model of normal and abnormal endothelium images. CI, confidence interval; PPV, positive predictive value; NPV, negative predictive value; AUC, area under the receiver operating characteristic curve.

### 3.2. Comparison between the performance of DCNNs and ophthalmologists

For 580 images tested independently, the average accuracy of the model, specialists, and senior physicians in distinguishing corneal layers was 0.974, 0.961, and 0.919, the average accuracy of identifying normal and abnormal images was 0.953, 0.954, and 0.907, the average time spent on 580 images was 12.80, 4520.00, and 9026.00 s, and the average time spent on each image was 0.02, 7.79, and 15.56 s, respectively. The accuracy of the model in distinguishing corneal layers and identifying normal and abnormal images was similar to that of two specialists (*p* = 0.872, >0.05) and higher than that of two senior physicians (*p* < 0.001). The evaluation speed was significantly faster than that of 4 ophthalmologists, which was about 390 times higher than that of specialists (Table 2).

**Table 2:** Comparison of the performance of the model and ophthalmologists.

		Accuracy of classification (95% CI)				
		Model	Expert A	Expert B	Senior doctor A	Senior doctor B
Epithelium	Layer	0.960 (0.928–0.992)	0.960 (0.928–0.992)	0.927 (0.884–0.969)	0.853 (0.796–0.911)	0.860 (0.804–0.916)
	Normal/abnormal	0.980 (0.957–1.003)	0.973 (0.947–0.999)	0.920 (0.876–0.964)	0.953 (0.919–0.987)	0.920 (0.876–0.964)
Bowman’s membrane	Layer	0.979 (0.956–1.003)	1.000	0.959 (0.926–0.991)	0.897 (0.846–0.947)	0.959 (0.926–0.991)
	Normal/abnormal	0.931 (0.889–0.973)	0.952 (0.916–0.987)	0.966 (0.935–0.996)	0.938 (0.898–0.978)	0.945 (0.907–0.982)
Stroma	Layer	0.987 (0.969–1.005)	0.961 (0.931–0.992)	0.955 (0.922–0.988)	0.955 (0.922–0.988)	0.884 (0.833–0.935)
	Normal/abnormal	0.929 (0.888–0.970)	0.968 (0.940–0.996)	0.935 (0.896–0.975)	0.839 (0.780–0.897)	0.787 (0.722–0.852)
Endothelium	Layer	0.969 (0.939–0.999)	0.954 (0.917–0.990)	0.969 (0.939–0.999)	0.985 (0.963–1.006)	0.977 (0.951–1.003)
	Normal/abnormal	0.977 (0.951–1.003)	0.954 (0.917–0.990)	0.962 (0.928–0.995)	0.985 (0.963–1.006)	0.908 (0.857–0.958)
Layer recognition average		0.974 (0.961–0.987)	0.969 (0.955–0.983)	0.952 (0.934–0.969)	0.921 (0.899–0.943)	0.917 (0.895–0.940)
Normal/abnormal classification average		0.953 (0.936–0.971)	0.962 (0.946–0.978)	0.945 (0.926–0.963)	0.926 (0.904–0.947)	0.888 (0.862–0.914)
Total		0.929 (0.908–0.950)	0.933 (0.912–0.953)	0.922 (0.901–0.944)	0.852 (0.823–0.881)	0.829 (0.799–0.860)

### 3.3. Comparison of the performance of the ophthalmologists with and without the model assistance

**Figure 4:**
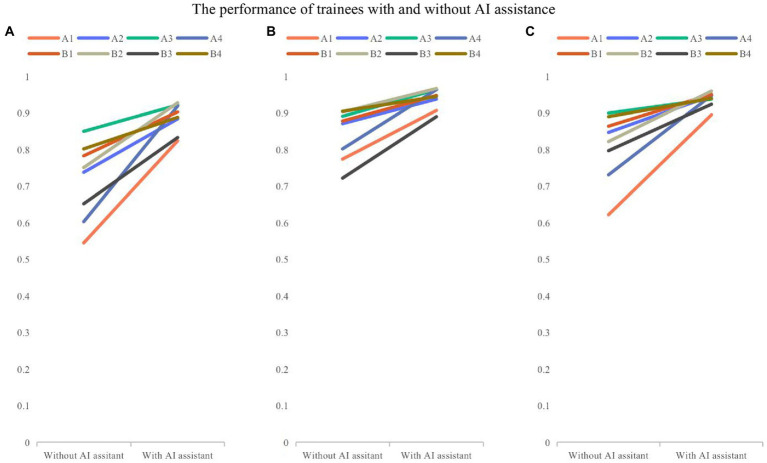
The comparison of performance of trainees with and without model assistance. (**A)** Overall accuracy; (**B)** the accuracy for recognizing layers; (**C)** the accuracy for classifying the normal and abnormal images. Folded-lines depict the changes in accuracy for each trainee with and without model assistance. Different colors represent different trainees.

The overall accuracy of 8 trainees with and without the model assistance was 0.888 and 0.715, respectively. Trainees had an accuracy of 0.843 and 0.809 without model assistance and 0.940 and 0.938 with model assistance in layer identification and classification of images as normal and abnormal. Both groups of trainees showed statistically significant differences in the accuracy with model assistance (0.17, *p* = 0.002, <0.05), and the changes in the accuracy of trainees are shown in Figure 4.

## 4. Discussion

In the present study, a model was developed based on 6 DCNNs to assess the capability of AI in distinguishing the layers of corneal IVCM images and classifying them as normal and abnormal. The results showed that the model was highly effective in distinguishing epithelium, bowman’s membrane, stroma, and endothelium, while it also had satisfactory accuracy, specificity, and sensitivity in distinguishing normal and abnormal images at each layer. The higher accuracy in some of the external test datasets than that of internal ones may be related to the slightly higher quality of external images, while data were retrospectively collected. A human-machine competition demonstrated that the model was as accurate as a corneal specialist and about 237 times as fast as a clinician. At the same time, the accuracy of IVCM image recognition by trainees with the model assistance could be significantly improved, and may even approach specialists’ accuracy, indicating that the model is potentially used to facilitate evaluation of IVCM images, and it is expected to facilitate the initial screening and classification of large volumes of images in actual clinical research, which can benefit physicians to promptly and centrally assess patients’ abnormal images or uniformly acquire specific layers and types of images, particularly for research purposes.

Due to the rapid progress of technology analysis, the improvement of computing power, and the promotion of big data, AI has recently developed rapidly in the field of healthcare (18, 19). Deep learning is one of the novel AI technologies, in which convolutional neural networks (CNNs) are programmed to optimize a specific performance criterion (20). In contrast to conventional machine learning methods, such as support vector machine, the deep learning-based methods allow the machine to identify complex features using hundreds of filters and eliminate the need for manual feature selection and extraction (21, 22). Deep learning has shown an outstanding performance in image recognition of skin diseases, cardiovascular system, respiratory system, digestive system, and other organs and diverse types of diseases (23). It has caused great changes in various diagnostic fields, including endoscopic ultrasound technology. At the same time, the accuracy of deep learning models in diagnosis and treatment of diseases also showed the potential of approaching or even surpassing physicians (24, 25).

It is widely accepted that AI has been widely used in the diagnosis, identification, and prevention of ophthalmic diseases (26). The United States Food and Drug Administration (FDA) also approved the first automatic diagnosis tool for diabetic retinopathy based on AI in 2018 (27). At the same time, AI has made remarkable achievements in the diagnosis, segmentation, and quantification of slit lamp images, anterior segment optical coherence tomography (A-S OCT), macular OCT, fundus fluorescein angiography (FFA), and other ophthalmic anterior and posterior segment images (11). IVCM photography and examination are well-established diagnostic imaging techniques for corneal diseases, while in clinical conditions, ophthalmologists mainly analyze images for multiple times to ensure accuracy of diagnosis. In the IVCM image recognition, there have been studies on AI-assisted quantification and segmentation of corneal nerves (14), classification of nerve fiber curvature (28), identification of nerve fibers and dendritic cells and fungal hyphae (12), discrimination of activated dendritic cells and inflammatory cells (9), segmentation of corneal endothelial cells, and evaluation of morphological parameters (15), fully highlighting that AI is used to assist IVCM image recognition to explore multiple structures. However, to our knowledge, cornea is a tissue with multiple layers, and few studies have concentrated on automatic multiple-layer corneal recognition even using traditional digital image analysis techniques, therefore, developing a more extensive tool to evaluate the cornea as thoroughly as possible can bridge the gap in this research area.

Res-Net-50 is a type of deep neural network that is a subclass of CNNs and is used to classify images, possessing the advantages of low computational burden and easy optimization. It contains 49 convolutional layers and a full connection layer, which are excellent residual network models. The residual network models can be used to solve the degradation and gradient problems, so that the network performance can be improved (29). According to the auxiliary clinical diagnosis and scientific research, 8 types of IVCM images (normal epithelium, abnormal epithelium, normal anterior elastic layer, abnormal anterior elastic layer, normal matrix, abnormal matrix, normal endothelium, and abnormal endothelium) were identified and diagnosed using one three-classification and five two-classification models, which were set up by Res-Net-50 in the present study. Firstly, DCNN1 and DCNN2 are used for corneal image level recognition, and then, DCNN3, DCNN4, DCNN5, and DCNN6 are utilized for normal and abnormal image recognition at each layer. Although the network is applicable to the diagnosis and recognition of a single image rather than a single patient, in clinical practice, examiners may collect dozens to hundreds of images for each patient. As long as all images of a single patient are imported into the model, the hierarchy and positive anomalies of the collected images can be obtained for IVCM of the patient.

In machine-aided image recognition, the accuracy of trainees in identifying corneal layers and abnormal image resolution has significantly improved with the development of models, indicating that the model proposed in the current study can also assist junior doctors to more reliably diagnose various abnormal corneal images and reduce the missed diagnoses of abnormal images. In the human-machine competition, the machine showed an accuracy similar to that of senior physicians, and the time spent was significantly shortened, demonstrating that the model may reduce physicians’ workload in actual clinical conditions, alleviate the influence of fatigue on the examination results, and is also worthy of promotion.

The present study has some limitations. Firstly, as the results achieved by the model for image recognition were qualitative, the actual abnormal features (e.g., cortical inflammatory cells, stromal hyphae, and endothelial KP) cannot be evaluated quantitatively or hierarchically. The enlargement of the training sample size in the next step of research is therefore suggested to more reliably segment and identify different abnormal features. Secondly, although as many IVCM images of clinical corneal diseases were collected as possible, due to certain requirements for sample data in model training, some rare corneal diseases were not included. Moreover, the number of images of few diseases involved in model development was relatively limited, which temporarily hindered further identification of types of disease for abnormal images. Hence, it will be attempted to cooperate with multiple hospitals to establish a database with involvement of more types of disease and a larger sample size for model training and optimization in the future, which will be advantageous to comprehensively analyze physicians’ IVCM image performance at all layers, enhance the diagnosis of various types of disease, and improve the accuracy of the model. It is suggested to apply AI to clinical and scientific research and facilitate the popularization of ophthalmic intelligent medical treatment.

In conclusion, a corneal IVCM image recognition model was developed based on deep learning. The results showed that the model had high accuracy, specificity, and sensitivity, and assisted clinicians to distinguish corneal IVCM images faster and more reliably. The model can be applied to communities and grassroots hospitals with little clinical experience or a lack of ophthalmologists, which can help novice doctors to identify and learn corneal IVCM images initially; it can also help professional doctors with heavy workload to screen images and quickly find the abnormal ones that need to be focused on. It is beneficial for preliminary screening of corneal diseases in large quantities of patients and obtaining specific corneal hierarchical images, particularly for research purposes, and lays the foundation for further building corneal disease identification models.

## Data availability statement

The original contributions presented in the study are included in the article/Supplementary material, further inquiries can be directed to the corresponding author.

## Ethics statement

The studies involving human participants were reviewed and approved by The Ethics Committee of Renmin Hospital of Wuhan University (Approval no. WDRY2021-K148). Written informed consent for participation was not required for this study in accordance with the national legislation and the institutional requirements.

## Author contributions

YuY, WJ, YZ, and YiY contributed to conception and design of the study. YuY performed the statistical analysis, designed the experiment, and wrote the first draft of the manuscript. BZ, HW, and LiH contributed to organized the database. LinH, SW, MT, yujingW, and HZ participated in the collection of experimental images. SC, YG, JM, yujingW, YC, QD, XS, ZY, and QM participated in the test of model performance. All authors contributed to the article and approved the submitted version.

## Funding

This work was supported by the grant from: The National Natural Science Foundation of China (no. 81770899 to YaY); The National Natural Science Foundation of China (no. 82101081 to SW); The Major Research Project of Hubei Province (no. 2020BCB055 to YaY).

## Conflict of interest

The authors declare that the research was conducted in the absence of any commercial or financial relationships that could be construed as a potential conflict of interest.

## Publisher’s note

All claims expressed in this article are solely those of the authors and do not necessarily represent those of their affiliated organizations, or those of the publisher, the editors and the reviewers. Any product that may be evaluated in this article, or claim that may be made by its manufacturer, is not guaranteed or endorsed by the publisher.
