# Supplementary material for: Evaluation of a computer-aided diagnostic model for corneal diseases by analyzing in vivo confocal microscopy images
Source: Front Med (Lausanne). 2023 Apr 20;10:1164188. doi: 10.3389/fmed.2023.1164188 (PMC10157182; doi:10.3389/fmed.2023.1164188)
Supplement: Supplementary file 1 [file Table_1.docx]

Table S1 Characteristics of subjects

| **Variables** | **Values** |
| --- | --- |
| **Training and internal test datasets** |  |
| No. of patients | 215 (training dataset) /53 (test dataset) |
| No. of images | 13277 (training dataset) /4245 (test dataset) |
| Male, n (%) | 113 (42.16%) |
| Age (years), mean (SD) | 48.53±15.94(19-72) |
|  |  |
| **Real-world external test datasets** |  |
| No. of patients | 109 |
| No. of images | 1510 |
| Male, n (%) | 49(45.37%) |
| Age (years), mean (SD) | 42.86±13.42(21-67) |
