# Supplementary material for: Evaluation of a computer-aided diagnostic model for corneal diseases by analyzing in vivo confocal microscopy images
Source: Front Med (Lausanne). 2023 Apr 20;10:1164188. doi: 10.3389/fmed.2023.1164188 (PMC10157182; doi:10.3389/fmed.2023.1164188)
Supplement: Supplementary file 2 [file Table_2.docx]

Table S2 Clinical diagnosis and number of included cases

| Image source | Number of patients with normal and various types of corneal diseases | | | | | | | | |
| --- | --- | --- | --- | --- | --- | --- | --- | --- | --- |
|  | Normal | Infectious keratitis | Moderate to severe dry eye | Fuchs corneal dystrophy | Anterior uveitis | Neurotrophic keratitis | Post corneal transplantation | Corneal chemical injury | Others |
| Renmin hospital  of Wuhan university | 128 | 89 | 32 | 27 | 23 | 18 | 13 | 12 | 57 |
| Zhongnan hospital of Wuhan university | 29 | 21 | 15 | 8 | 7 | 8 | 5 | 4 | 11 |
